# Short term dynamics of the sputum microbiome among COPD patients

**DOI:** 10.1371/journal.pone.0191499

**Published:** 2018-03-08

**Authors:** Rohita Sinha, Lisa A. Weissenburger-Moser, Jennifer L. Clarke, Lynette M. Smith, Art J. Heires, Debra J. Romberger, Tricia D. LeVan

**Affiliations:** 1 University of Nebraska, Department of Food Science & Technology, Lincoln, NE, United States of America; 2 University of Nebraska Medical Center, Department of Epidemiology, Omaha, NE, United States of America; 3 University of Nebraska Medical Center, Department of Biostatistics, Omaha, NE, United States of America; 4 University of Nebraska Medical Center, Department of Internal Medicine, Omaha, NE, United States of America; 5 University of Nebraska Medical Center, Department of Internal Medicine and Veterans Nebraska Western Iowa Healthcare System, Omaha, NE, United States of America; Lee Kong Chian School of Medicine, SINGAPORE

## Abstract

Chronic obstructive pulmonary disease (COPD) is an inflammatory disorder characterized by incompletely reversible airflow obstruction. The complexity of the lung microbial community in COPD patients has been highlighted in recent years. Evidence suggests that transplantation, medications, age, and disease severity influence microbial community membership. However, the dynamics of the lung microbiome in stable COPD patients remain poorly understood. In this study, we completed a longitudinal 16S ribosomal RNA survey of the lung microbiome on replicate sputum samples collected from 4 former smokers with COPD (Stage 2) within a 2-day time period. Samples from each individual over the two-day period were similar based on α-diversity, principle component analysis and taxonomy at the phyla and genera level. Sputum samples from COPD patients were also collected between 2–9 months of follow-up. Data suggest an increased variability of the sputum microbiota when comparing samples collected ≤ 3 months compared to those collected ≥ 4 months; however, no statistically significant shifts in the abundance (>2-fold) of taxa between the two time points was observed. Bacterial composition and the number of operational taxonomic units (OTUs) remained similar over time. Results from this study suggest that the sputum microbiome is relatively stable in clinically stable COPD patients (Stage 2). This study furthers our understanding of the dynamics of the lung microbiome in COPD patients.

## Introduction

Chronic obstructive pulmonary disease (COPD), a disease characterized by persistent airflow limitation and chronic inflammation [1], is the third-leading cause of death in the United States. Although tobacco smoke is the leading risk factor for COPD worldwide, other exposures such as agricultural dust, biomass fuel smoke and air pollution contribute significantly in disease prevalence [2,3].

There has been controversy regarding the role of lower respiratory tract bacteria in COPD pathogenesis [4]. It was believed that the lungs of healthy individuals were sterile. However, since the first report of a lung microbiome, many studies have found evidence of bacteria in the lower airways using new culture-independent methods [5–8]. The core airway microbiome in the healthy lung is predominantly comprised of the phyla Bacteriodetes, Firmicutes and Proteobacteria [7,9,10]. This community is modified in individuals with lung disease [9–11]. Many patients with COPD during clinical stability show evidence of chronic bacterial colonization with pathogens of the phylum Proteobacteria including *Haemophilus* spp. and *Moraxella* spp. [8,12,13], while *Bacteroidetes* were more prevalent in control patients [8].

Respiratory viral infections, especially rhinoviruses, are a major cause of COPD exacerbations [14]. Molyneaux et al. investigated the effect of rhinovirus infection on the airway bacterial microbiome and discovered that rhinovirus infection in COPD alters the respiratory microbiome [15]. An increase in Proteobacteria, most notably in *Haemophilis*, was observed in patients with the viral infection compared to healthy individuals.

Few longitudinal studies examining the change of the lung microbiome over time have been conducted [16–18]. One study examined the lung microbiome during acute exacerbations and found lower abundances of genera *Moraxella* and *Streptococcus* in sputum samples compared with samples taken during clinical stability [16]. Another study identified increases in *Haemophilus*, *Pseudomonas*, *and Moraxella* during exacerbations compared to paired sampling from periods of clinical stability in COPD patients [17]. All of these studies have shown that changes in the lung microbiome in patients with COPD occur when there are exacerbations and respiratory infections [19–21]. As yet, there are no longitudinal studies comparing baseline sputum samples with samples collected over time in clinically stable COPD patients.

The aim of this study was to examine the microbiome longitudinally in individuals with stable COPD following 16s rRNA gene sequencing of sputum. We hypothesized that the microbiome of COPD patients is relatively stable within a short period of time. We collected replicate induced sputum samples from four patients with stable COPD (Stage 2) over a two-day period and examined the community variability within each patient. In addition, we assessed the variability of the microbiome at baseline and between 2 to 9 months for 7 patients, and determined whether the microbial community changed over this time period.

## Materials and methods

### Patient recruitment

The AgLung cohort is a cross sectional study of agricultural exposures and respiratory disease among veterans visiting the General Medicine clinics at the VA Medical Center in Omaha, NE [22]. Other than working on a farm for more than two years, eligibility criteria included being between 40 and 80 years of age and no history of lung cancer, metastatic cancer to the lungs or interstitial lung disease such as pulmonary fibrosis, asthma, sarcoidosis, and hypersensitivity pneumonitis. A sub-group of COPD patients from the AgLung cohort was included in the present analysis. Patients eligible for this study were former smokers and had COPD (Stage 2), COPD was defined as post-bronchodilator forced expiratory volume in one second (FEV_1_)/forced vital capacity (FVC) ratio ≤ 0.7, 50–79% predicted FEV_1_ and/or a diagnosis of COPD from a pulmonologist. Eligible individuals had stable respiratory symptoms, were afebrile, and had not taken any antibiotics or corticosteroids for two months prior to sputum induction. This research was approved by the VA Nebraska-Western Iowa Health Care System IRB and written informed consent was obtained from all subjects.

### Sputum collection

For the two-day study, replicate samples from four patients over a two-day period were collected using an established induced sputum protocol developed by the NIH-sponsored SPIROMICS study for COPD [23]. Briefly, three 7-minute inhalations of nebulized hypertonic saline (3%) were followed by expectoration of the sputum. We acknowledge that using induced sputum for inference of the lung microbiome has its drawbacks, including oral contamination. To minimize oral contamination, all subjects performed an oral rinse with mouthwash (Cepacol, Reckitt Benckiser, Parsippany, NJ, USA), sterile water, then molecular grade water prior to obtaining an induced sputum sample.

A baseline sample was collected in the morning on day one, another 4–5 hours later, and then again the next morning. One patient gave a fourth sample in the afternoon on the second day.

For the 9-month study, two induced sputum samples were obtained, one at baseline and another within nine months of follow-up. Participants went through the same three 7-minute inhalation protocol as described above. Individuals with the ID’s 2195, 2150 and 1204 participated in both the 2-day and 9-month study.

### Sputum processing, DNA extraction, PCR amplification, and pyrosequencing

Sputum was processed by a modification of the method developed by Alexis et al. [24]. Briefly, freshly-collected sputum was weighed and 0.9 mL of the sample including all mucous plugs was solubilized in 0.1% dithiothreitol, diluted 4-fold with EDTA, and filtered through a 0.48 mm mesh strainer. Cells isolated from this fraction were cyto-centrifuged, stained using a Wright’s-Giemsa histologic stain (Diff-Quik, Fisher Sci. St. Louis, MO), assessed microscopically using a Neubauer hemacytometer (four high-power fields or 400 cells counted per sample) and macrophages, neutrophils and epithelial cells quantified. Squamous cell number was recorded as an indicator of the sputum sample quality. Greater than 40% squamous contamination (from the mouth and throat) excluded the sample. DNA was isolated from the remaining solubilized sputum using a bead beating, solvent extraction method (PowerSoil DNA isolation Kit, Mo Bio) according to the manufacturer’s instructions. An approximately 460 bp sized fragment of the V3 / V4 region of the 16S rRNA gene was amplified (25 cycles of PCR) for each of the DNA samples beginning with 12.5 ng of DNA per Illumina’s recommended protocol outlined in the 16S Metagenomic Sequencing Library Preparation protocol (Illumina, San Diego, CA). 16S Amplicon PCR Forward Primer = 5'TCGTCGGCAGCGTCAGATGTGTATAAGAGACAGCCTACGGGNGGCWGCAG 16S Amplicon PCR Reverse Primer = 5'GTCTCGTGGGCTCGGAGATGTGTATAAGAGACAGGACTACHVGGGTATCTAATCC. Following generation of the amplicons, dual indices and Illumina sequencing adapters were added (8 cycles of PCR) using the Nextera XT Index kit (Illumina catalog # FC‐131‐1001). Resultant libraries were multiplexed and 300 bp long, paired-end sequence data was generated on an Illumina MiSeq instrument using V3 chemistry per Illumina’s recommendations. An ample yield of at least 4,000 reads/sample was used to characterize the bacterial community from each sample. The use of at least 4000 reads/sample as a threshold is appropriate in this study and reflects the microbial diversity in our samples because the rarefaction curves plateaued at 1500–2000 reads/sample (S1 and S3 Figs).

### Data processing and bioinformatics analysis

Samples with paired-end Illumina reads were filtered to trim the low quality bases from 3' as well as 5' ends using Cutadapt package1.2.1 [25]. Given a significantly lower quality of the bases at 3' end of the reads, the quality-value (Phred Score) cutoffs for 3' and 5' bases were 35 and 30, respectively. Quality-trimmed read pairs with the minimum length of 150bp were retained, and used in the subsequent analysis performed using Illumina Base-Space cloud platform for processing the 16S rRNA gene data (version 2.0.5.35.6). The sequence data has been deposited in the NCBI BioSample database and can be accessed via the following link https://www.ncbi.nlm.nih.gov/Traces/study/?acc=SRP124904.

In the downstream analysis, default Qiime parameters for Preprocessing and Visualization apps were used [26]. Post-QC reads were aligned against the Greengene database [27]. The number of sequences excluded due to not aligning with the Greengenes database is presented in S1 and S2 Tables. Following the taxonomy assignment, the number of sequences assigned to a particular phylotype and the percentage of these sequences in the microbial community were calculated for each sample. Samples with less than 4,000 total cleaned reads were excluded. Reads with more than 97% identity were tallied to make the counts and percentages tables, with each row representing a different phylotype. Alpha (Shannon and Chao1 Indices) and Beta (Unifrac weighted) diversity scores were calculated. Unifrac distance metric was used for performing the Principal Component Analysis (PCoA) plots. Community membership and structure were examined using the PCoA plots to determine relatedness among samples. Heat maps showing similarity or dissimilarity among samples were also generated.

### Statistical analysis

For the two-day study, patients were seen at least three times: Day 1, morning (D1_1); Day 1, afternoon (D1_2); Day 2, morning (D2_1); and one patient was seen Day 2, afternoon (D2_2). We evaluated the Shannon and Chao1 indices (Wilcoxin Signed-Rank Test), PCoA plots, relative abundance of taxa and rarefaction curves for each subject during the two-day period.

For the 9-month study, samples from subjects were defined according to the time when the sputum sample was taken, baseline (T1) versus months later (T2, range 2 to 9 months). The taxon-based method was used to analyze the 16S rRNA sequence of each sample as described above. We compared the Shannon and Chao1 indices (Wilcoxin Signed-Rank Test), PCoA plots, relative abundance of taxa and rarefaction curves for each subject at T1 and T2. Differences in percent (%) of taxonomic abundance, T2 minus T1, were calculated.

The DEseq2 algorithm was used to compare the prevalence of the taxa between the two sample groups (T1 vs. T2) as follows. At different taxonomic levels, the relative abundance was compared by the DESeq2 method to identify the taxa among the groups, while accounting for the paired nature of the data [26]. The Benjamini and Hochberg false discovery rate (FDR) approach was used to adjust the raw p-values to account for multiple comparisons [28]. Taxa yielding an FDR adjusted p-value < 0.05 were considered statistically significant.

## Results

### Two-day study

We examined the variability of the sputum microbiome for four COPD patients over a two-day period. All patients were seen at least three times. Sample D2_1 for patient ID 1204 had very low sequencing reads (<4,000), and therefore was excluded from the analysis. Patient demographics are summarized in Table 1. The mean age of the participants was 63 years (± 7.4 SD) and had worked on a farm for an average of 35 years (± 20 SD). The mean FEV_1_/FVC ratio and % predicted FEV_1_ was 0.61 (± 0.1 SD) and 60.7 (± 7.7 SD), respectively. The number of OTUs detected at 97% identity was on average 375 (± 103 SD) for the 12 samples (Table 1). There were no significant differences in the number of OTUs obtained from day 1 and day 2 (p = 0.61, Mann-Whitney U Test). We compared the Shannon diversity indices for each patient at each time point using the Wilcoxin Signed-Rank Test. However, the N (5) was not large enough for the distribution of the statistic to form a normal distribution thus an accurate p-value could not be calculated. On average we observed a 3% (± 0.5 SD) change in the Shannon Index between day 1 and day 2 samples (Table 1).

**Table 1:** Patient characteristics and sequencing results: 2-day study.

Sample ID	Sputum Induction	Age	FEV_1_ % Predicted	FEV_1_/FVC	BMI	Farm Work (yrs)	OTUs^	Chao1 Index*	Shannon Index*
1204-D1_1	Day 1	67	64	0.62	≥30	48	171	235.3	5.31
1204-D1_2	Day 1						252	265.0	5.52
2108-D1_1	Day 1	70	51	0.53	≥30	56	379	368.1	6.04
2108-D1_2	Day 1						479	438.9	5.83
2108-D2_1	Day 2						453	366.8	6.10
2150-D1_1	Day 1	53	59	0.67	≥30	14	313	232.7	4.91
2150-D1_2	Day 1						318	234.0	5.05
2150-D2_1	Day 2						347	298.0	5.22
2195-D1_1	Day 1	62	69	0.60	≥30	21	447	409.6	5.83
2195-D1_2	Day 1						541	423.9	6.07
2195-D2_1	Day 2						375	371.0	5.91
2195-D2_2	Day 2						427	338.1	5.66

*Abbreviations and Definitions*: FEV_1_, % predicted forced expiration volume in 1 second (L); FEV_1_/FVC, ratio of forced expiratory volume in 1 second/forced vital capacity (L); BMI, body mass index (kg/m^2^), OTU, Operational Taxonomic Units.

^Number of OTUs

*Alpha diversity based on the evenly subsampled data (4,000 reads / sample)

**Fig 1:**
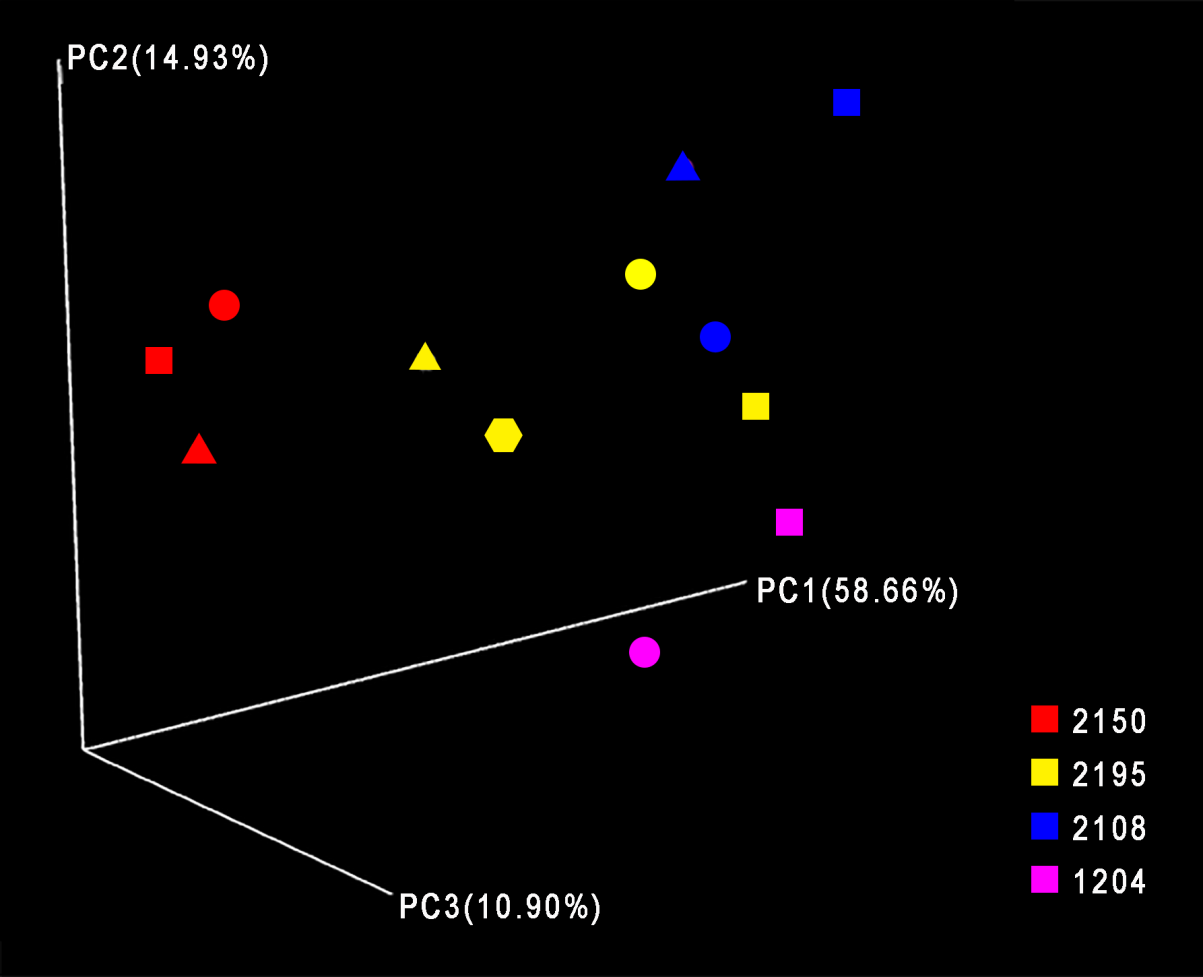
Principal coordinate analysis demonstrates clustering of two-day study samples. Principal coordinate analysis was performed using Qiime version 1.9.1-dev and Weighted UniFrac, and the results for principal coordinates 1, 2 and 3 are shown. Square = D1-1; Circle = D1-2; Triangle = D2-1; Hexagon = D2-2.

Rarefaction curves showed that additional sequencing depth would not have provided detection of additional OTUs (S1 Fig). Using heat maps, PCoA and the UniFrac distance metric, we observed tight clustering of samples taken from the same patient (S2 Fig and Fig 1). These results suggest there is little variability of the sputum microbiome over a two-day period in these COPD patients.

**Fig 2:**
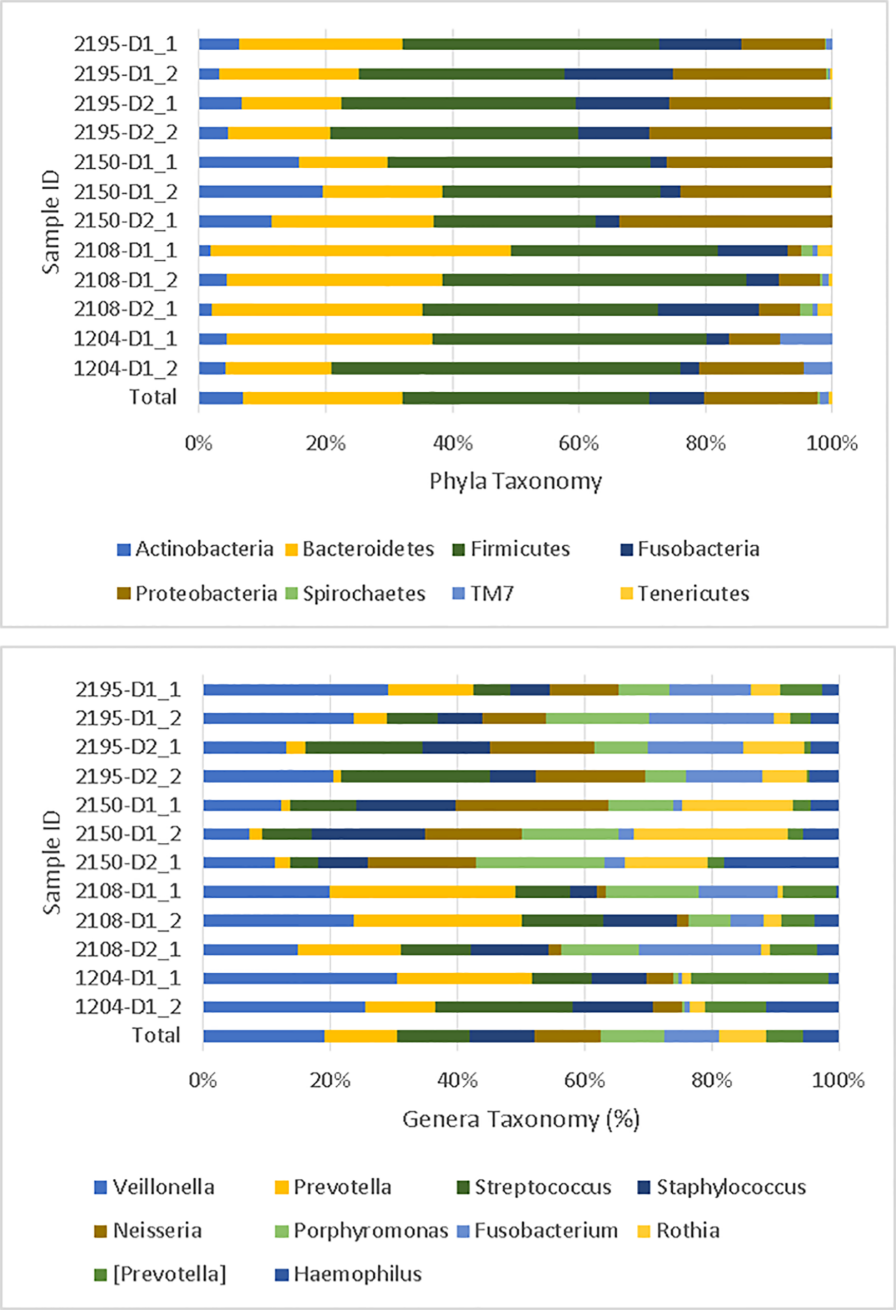
Taxonomic identification for two-day study samples. *Top*. Taxonomic results at the phylum level are displayed for each sample. *Bottom*. Taxonomic identification for the top ten genera for each sample.

Sequences were submitted to the Greengenes database for taxonomic identification. Phylum-level classification for each sample is provided in Fig 2 (*Top*). Approximately 90% of sequences belonged to one of four phyla: Firmicutes (38.9%), Bacteroidetes (25.1%), Proteobacteria (17.9%), and Actinobacteria (7.0%). At the genera level, *Veillonella* (14.8%) was the most abundant, followed by *Prevotella* (8.8%), *Streptococcus* (8.8%), and *Rothia* (5.7%) (Fig 2, *Bottom*). *Haemophilus* was the 10^th^ top genera among these samples (4.3%). Because our sample size was very small, we could not effectively use the DEseq2 method to detect differentially abundance features between samples for each individual.

### Nine-month study

Sputum samples from seven subjects were obtained at baseline (T1) and at follow-up (T2, ≤ 9 months). Patient demographics are summarized in Table 2. The majority of the participants (87.5%) was over the age of 60 and worked on the farm for greater than 20 years (62.5%). The mean FEV_1_/FVC ratio and % predicted FEV_1_ was 0.67 (± 0.04 SD) and 64.0 (± 7.3 SD), respectively. DNA sequencing reads (337,474) were selected after demultiplexing and quality control filtering, with each sample averaging 24,105 trimmed sequences. The average number of unique OTUs identified across 14 samples was 456 (± 130 SD). There were no significant differences in the number of OTUs obtained from baseline and at the later time point (p = 0.70, Mann-Whitney U Test). We used the Wilcoxin Signed-Rank Test to compare the Shannon index for a subject at each time point. The size of the N (7) was not large enough for the Wilcoxin statistic to form a normal distribution; therefore, it was not possible to calculate an accurate p value. There was on average a 3.6% (± 5.4 SD) change in the Shannon index for samples collected ≤ 3 months compared to a 10.2% (± 6.7 SD) change for samples collected ≥ 4 months.

**Table 2:** Patient characteristics and sequencing results: ≤ 9 month study.

Sample ID	Sputum Induction	Age	FEV_1_ _%_ Predicted	FEV_1_/FVC	BMI	Farm Work (yrs)	OTUs^	Chao1 Index*	Shannon Index*
2195-T1	Baseline	62	69	0.60	≥30	21	607	541.8	6.05
2195-T2	2 months						585	564.8	6.04
2150-T1	Baseline	53	59	0.67	≥30	14	472	565.1	5.18
2150-T2	6 months						231	400.7	4.19
2326-T1	Baseline	70	69	0.70	≥30	43	420	558.4	5.78
2326-T2	2 months						620	604.2	6.58
1053-T1	Baseline	59	53	0.71	≥30	49	481	484.5	6.01
1053-T2	9 months						550	510.6	6.53
1204-T1	Baseline	67	64	0.62	≥30	48	309	425.1	5.31
1204-T2	3 months						225	283.0	5.24
2397-T1	Baseline	61	74	0.68	<25	20	473	558.1	6.05
2397-T2	4 months						396	516.3	6.27
2285-T1	Baseline	66	59	0.71	≥30	48	574	754.1	5.90
2285-T2	4 months						447	493.6	5.23

*Abbreviations and Definitions*: FEV_1_, % predicted forced expiration volume in 1 second; FEV_1_/FVC, ratio of forced expiratory volume in 1 second/forced vital capacity (L); BMI, body mass index (kg/m^2^), OTU, Operational Taxonomic Units.

^Number of OTUs.

*Alpha diversity based on the evenly subsampled data (10,000 reads / sample)

**Fig 3:**
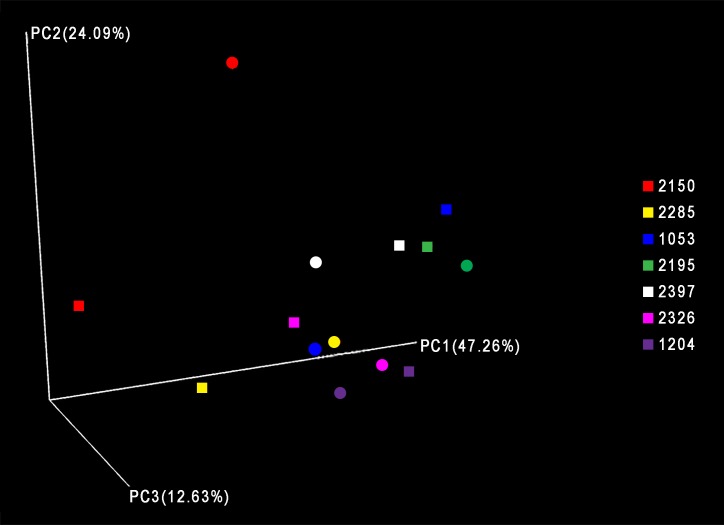
Principal coordinate analysis of 9-month study samples. Principal coordinate analysis was performed using Qiime version 1.9.1-dev and Weighted UniFrac, and the results for principal coordinates 1 and 2 and 3 are shown. Square = T2; Circle = T1.

Similar to the two-day study, rarefaction curves were calculated for all samples and maximum OTUs were detected, showing that additional sequencing depth would not have detected additional OTUs (S3 Fig). Using heat maps and PCoA with weighted UniFrac, we found clustering of samples taken from the same patient (Fig 3, S4 Fig). On visual inspection, the data suggest increased dispersion of clustering for samples collected ≤ 3 months compared samples collected ≥ 4 months.

Based on overall phyla composition, the samples were composed of four major groups: Proteobacteria, Firmicutes, Bacteroidetes, and Actinobacteria (S5 Fig, *Top*). Approximately 90.5% of sequences belonged to four phyla: Firmicutes (44.1%), Bacteroidetes (23.3%), Proteobacteria (12.3%), or Actinobacteria (10.8%). Of the 183 genera identified, the most abundant were *Veillonella* (17.2%), *Prevotella* (11.8%), *Streptococcus* (11.1%), *Rothia* (7.6%), and *Haemophilus* (3.9%), all of which are typical members of the lung microbiota [29] (S5 Fig, *Bottom*). These top ten genera were 100% prevalent across all samples from both visits (T1 and T2), suggesting that for our COPD cohort, the sputum microbiome did not undergo major shifts in genera over this time period [30].

**Fig 4:**
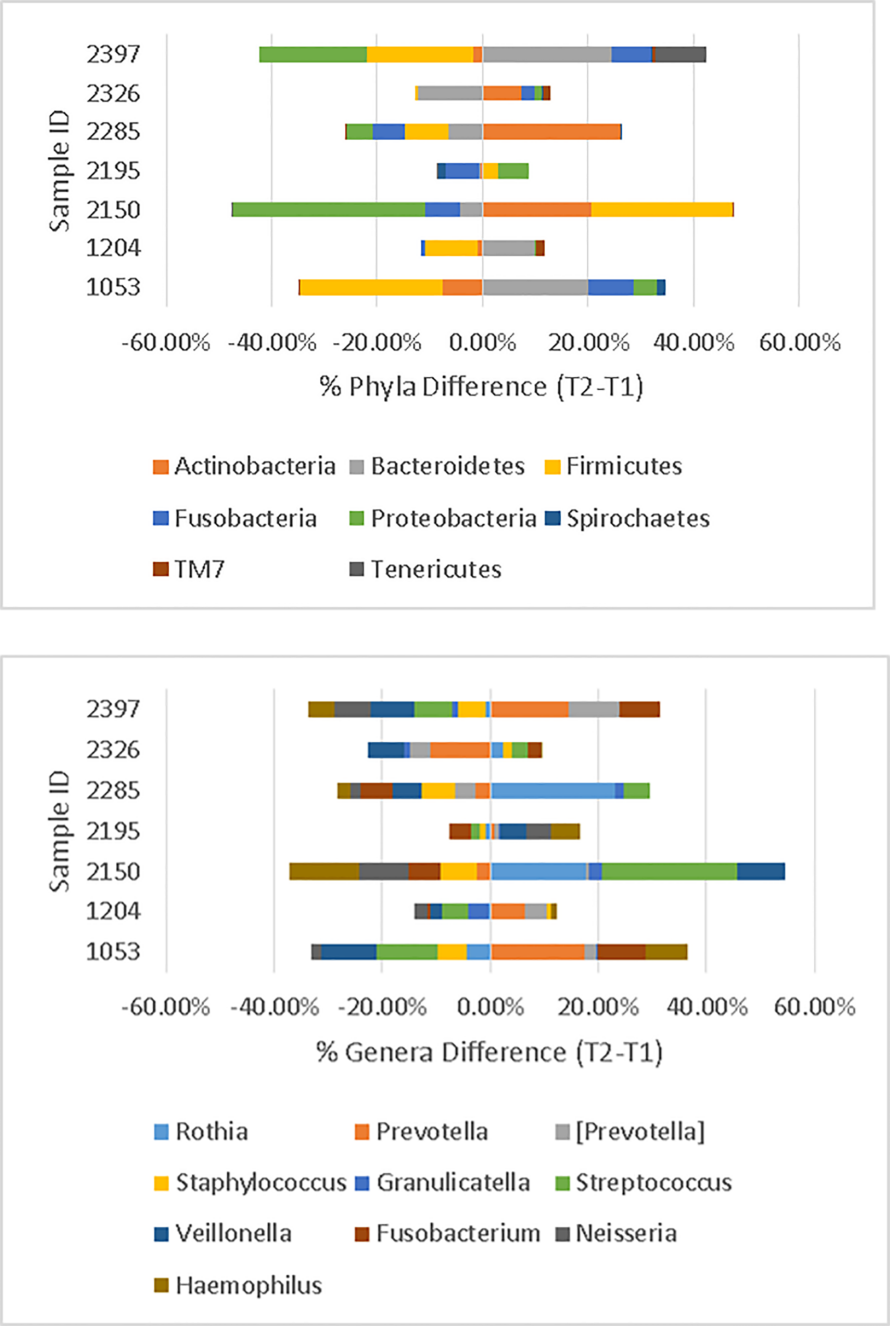
*Top*. Differences in percent of taxonomic abundances at the phylum level in the 9-month study (T2 minus T1). For example, patient 2150 had a 36.4% decrease in abundance of Proteobacteria from T1 to T2. *Bottom*. Differences in percent of taxonomic abundances at the Genus level (T2 minus T1). Top taxa are displayed. Figs were created in Excel2010.

The difference in phyla abundance between the two time points is presented in Fig 4 (*Top*). The largest change was found in patient ID 2150 (T2-T1 = 6 months). Actinobacteria changed from 8.2% to 28.9%, Firmicutes increased from 38.2% to 64.8%, and Proteobacteria dropped from 37.9% to 1.5%. For this patient, the Shannon Index (4.19) for the six-month sample was less diverse than the baseline sample (Shannon Index = 5.18). Furthermore, the phyla differences for T1-T2 samples taken ≤ 3 months apart appeared to change less than samples taken ≥4 months apart.

The difference in genera abundance between two time points is presented in Fig 4 (*Bottom*). Again, patient ID 2150 had the largest variations. *Rothia* increased to 25.8% from 8.0%, *Streptococcus* increased to 28.7% from 3.7%, and *Haemophilis* decreased to 0.6% from 13.4%. Of note, ID 2150 at T = 1 had the largest percentage of *Haemophilus* (13.4%) compared to the rest of the samples (S4 Fig).

**Fig 5:**
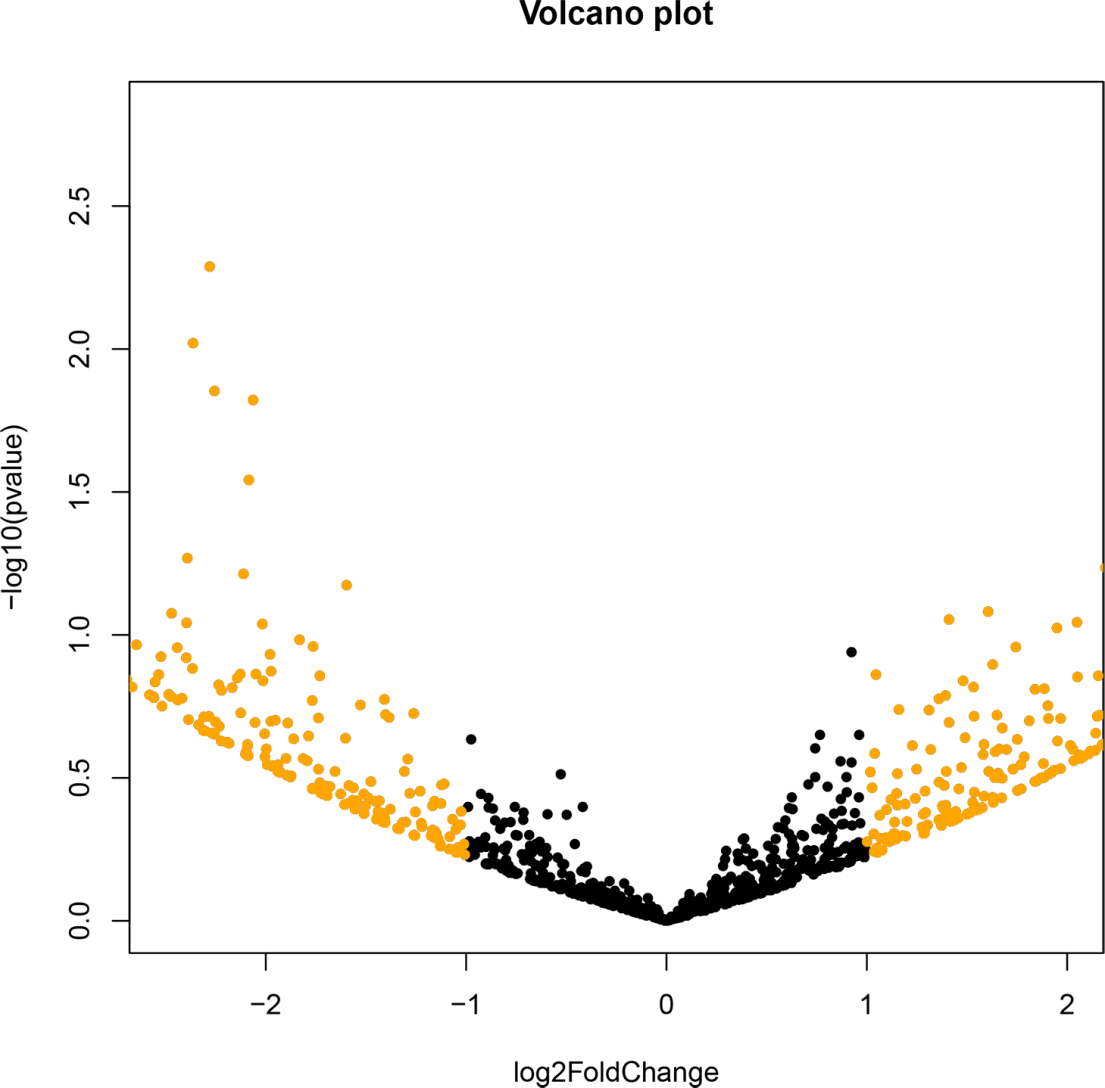
Volcano plot for the 9-month study. Pairwise comparisons using DESeq2 version 1.6.3). Results shown are from all seven participants. Color differences show relative abundance of at least 2-fold, or log_2_ equal to or less than 1 (black) versus greater than 1 (gold). Fig was created in R phyloseq, (version 1.10.0).

To determine if there were any differences between T1 and T2 taxa, we used the DEseq2 method to detect differentially abundant features between samples. For each taxa a p value was generated and zero OTUs were significantly different among the two groups (T1 and T2) (all p values > 0.05). To examine the microbiome stability at two time points, we used a volcano plot (Fig 5) and, again, determined there were no taxa that exhibited differential relative abundance, based both on visual inspection and statistical thresholds (p<0.05). However, there were several OTUs that had log2-fold change greater than 2 (indicated in gold) (S3 Table).

## Discussion

This study used induced sputum samples to explore the short-term stability of the lung microbiome in clinically stable COPD patients. We hypothesized that the sputum microbiome of individuals with COPD would have similar bacterial compositions and diversities during a short time period (≤ 2-days). Our data is consistent with this hypothesis and suggests a high degree of similarity of the sputum microbiome in COPD patients when sampled over 2 consecutive days. These results are in contrast to the airway microbiota composition of sputum when sampled over a longer period of time, where we found increased variability in samples collected ≥ 4 months from baseline.

The lung microbiome of healthy individuals has been shown to consist of bacteria from the Bacteriodetes, Firmicutes, Proteobacteria, and Actinobaterica phyla [12,31], which are all phyla found in our sputum samples. In this study, Firmicutes was the most prevalent phylum, a phylum primarily composed of gram-positive microorganisms, followed by Bacteriodetes. A study conducted by Hilty et al. showed Bacteroidetes, particularly *Prevotella* spp., was more prevalent in controls than in COPD patients [8], while Proteobacteria, a gram-negative phylum, was more prevalent in COPD patients compared to controls [8].

We demonstrated similar genera in COPD patients compared to prior studies including *Streptococcus*, *Prevotella*, *Moraxella*, and *Haemophilus* [4,32]. Different techniques such as expectorated sputum, bronchial aspirate, bronchoalveolar lavage (BAL), and bronchial mucosal biopsies, have all found these genera in COPD patients [32]. In our study, only *Streptococcus* and *Haemophilus*, not *Pseudomonas* or *Moraxella*, were represented in the top ten genera of the 14 samples. Of note, *Haemophilus* was most prevalent in 2150-T1, possibly due to complex medical issues of this patient as described below.

The lungs can be difficult to study using longitudinal and cross-sectional study designs. In previous studies, factors including interventions (transplantation and medications), age, exacerbations, and disease severity have been shown to influence microbial community membership [8,16,21,30,33]. We attempted to control for these covariates during the patient selection phase as described below. Patient ID 2150 showed the greatest differences in the bacterial composition between the two time points. This patient’s medical chart indicated multiple health issues including heart failure, chest pain, and arrhythmia. Although patient ID 2150 was not taking antibiotics two months prior to collection of induced sputum samples, he had taken clindamycin after the first sample and prior to the second sample. Antibiotics have been shown to increase microbial diversity, though this did not occur in our observations of this patient [20,30]. Our observations did show a large decrease in Proteobacteria and an increase in Firmicutes in this patient, which is in agreement with previous studies regarding antibiotic use among COPD patients [20,30]. Age has been shown to be associated with microbial diversity [33]. Pragman et al. showed younger age is associated with less microbial diversity in COPD patients [33]. Although patient ID 2150 was the youngest compared to others in this study, only his second sample showed the lowest diversity among all samples.

COPD is characterized by natural histories that are punctuated by periods of acute exacerbations. A study conducted by Sethi et al. identified lower densities of *Moraxella catarrhalis* and *Streptococcus pneumonia* in sputum samples collected during acute exacerbations compared with samples during clinical stability [16]. Another study examined sputum from COPD patients and identified increases in *Haemophilus*, *Pseudomonas*, and *Moraxella* during exacerbations compared to paired sampling from periods of clinical stability [17]. Both, Huang et al. and Molyneaux et al. found an increase in the phylum Proteobacteria during COPD exacerbation [21,30]. What’s interesting to note is that *Moraxella* was found in only one sample of this study, ID 2396 (time = 2) at 0.7%.

The relationship between bacterial diversity and COPD severity remains disputed. Studies have reported that bacterial diversity decreases with increased COPD severity [4,8]; however, other studies using lung tissue samples have failed to show significant differences in bacterial diversity with increasing COPD severity [10]. All of the patients in our study had Stage 2 COPD at the time of enrollment into the AgLung study; therefore, we were unable to investigate the relationship between COPD stage and bacterial diversity. Because this study only included patients with stable COPD, we cannot generalize our results to individuals without COPD. Although patient ID 2326 had a borderline diagnosis of COPD, the bacterial composition and diversity of his samples were similar to those with COPD stage two in this study. Notably, both of patient ID 2326’s samples clustered with all other samples (except ID 2150) on the PCoA plot.

This study only included former smokers as current smoking has been shown to have an impact on the lung microbiome in COPD patients [34]. Some believe that smoking in and of itself does not alter the lung microbial community [35]; however, current smokers were found to have lower 16S rRNA copy numbers. And while 16S rRNA copy number cannot be directly compared with measures of bacterial community diversity, as some bacteria can possess multiple copies of the 16S rRNA gene, some studies have shown smokers exhibit greater variation in the relative abundance and composition of bacteria inhabiting the nasal or oropharynx [36]. Hilty et al. examined the lung microbiome using airway brushings and included COPD patients that were current smokers (80%) [8]. They found Proteobacteria to be the most abundant phylum in COPD patients. Although Proteobacteria was one of the top four phyla in our study, Firmicutes was the most abundant. A handful of studies have evaluated the lung microbiome of COPD patients who smoke; however, additional studies with larger sample sizes are required to definitively determine if the lung microbiome is altered by smoking in those with COPD.

The assessment of the lung microbiome in agricultural workers has not been studied. However, shotgun pyrosequencing metagenomic analyses of DNA from dusts from swine confinement facilities and grain elevators have been performed [37]. Boissy et al. identified Firmicutes as the predominate phylum in dust from a swine confinement facility. In contrast, Proteobacteria was the most abundant phylum in grain elevator dust [37]. Approximately 71.5% of individuals in this study worked with hogs, either in confinement or open lots, in their lifetime, while 100% were exposed to grain dust. All patients were former farmers in this study; and therefore, most likely the influence of the agricultural environment did not play a role in influencing the lung microbiome unless there were long term effects. Further studies are needed to evaluate the effect of agricultural exposure on the lung microbiome in those with and without COPD.

To our knowledge, this study is the first to present a longitudinal analysis of the lung microbiome in patients with COPD during clinical stability. One of the admitted limitations of this study is the sample size, and therefore descriptive in nature. In fact, relatively small sample sizes have plagued many lung microbiome studies and most likely limited our ability to observe significant differences between our groups. Inducing sputum from patients is difficult and time-consuming and for safety reasons, we only induced sputum in those with COPD Stage 2. Collecting samples using induced sputum is a non-invasive alternative to bronchoalveolar lavage (BAL). Studies comparing the microbiome at different sites along the aerodigestive tract find that this body site constitutes a microbiological continuum with substantial species overlap but with varying abundances [6,7]. Consequently, it is challenging to define a distinct lung microbiome to filter out oral contamination from the induced sputum 16S data. Additionally, sputum samples have been shown to have significantly lower diversity than other sample types such as BAL, cell-free BAL supernatants, and biopsies [32].

In the ≤ 9 month study, the time between samples taken varied among our patients. Patient ID 1053 had the largest elapsed time period between induced sputum samples (9 months). In this patient, the phylum Firmicutes in T2 decreased from 54.5% to 27.5% and the genus *Prevotella* increased by approximately 27% from the baseline sample (T1 = 10.1%). Patient ID 2150 had approximately six months between the two time points and we saw a decrease in microbial diversity. However, in this patient we saw an increase in Firmicutes and a decrease in *Prevotella*. Additional longitudinal analyses with greater patient numbers are needed to help better understand the dynamics of the lung microbiome in relation to COPD. We were unable to control for the time intervals in this study due to difficulty in patient follow up. Coordinating with these patients was difficult as patients were busy, some became sick, and one patient had to cancel due to a sick family member.

In summary, we have presented the induced sputum bacterial profiles of agriculturally- exposed, male patients with moderate COPD based on 16S rRNA gene sequencing. We showed that the sputum microbiome remains stable over a 2-day period and with increasing variability over a 9-month period. This study adds further insights into the microbiome of COPD patients, with the inclusion of repeated and longitudinal sampling.

## Supporting information

S1 Fig Rarefaction curves for two-day study samples. X-axis represents sequences per sample. Y-axis is Shannon rarefraction measure. Legend shows the color representing each sample. Figure was created in Qiime version 1.9.1-dev.
(TIF)

S2 Fig Heat map of OTU abundances for two-day study samples. Lighter blue shows greater abundance compared to dark blue/black. Figure was created in R (phyloseq version 1.7.12) using all aligned OTUs.
(TIF)

S3 Fig Rarefaction curves for 9-month study samples. X-axis is sequences per sample. Y-axis is Shannon Rarefraction Measures. Legend shows the color representing each sample. Figure was created in Qiime version 1.9.1-dev.
(TIF)

S4 Fig *Top*. Heat map of OTU abundances at baseline (T1) and at the later time point (T2) in the 9-month Study. *Bottom*. Heat map of differences of OTU abundances between T1 and T2 (T2 minus T1). Lighter blue shows greater abundance difference compared to dark blue/black. Figures were created in R (phyloseq version 1.7.12) using all aligned OTUs.
(TIF)

S5 Fig *Top*. Taxonomic identification at the phylum level in the 9-month study. *Bottom*. Taxonomic identification at the Genus level. Taxonomic results at the phylum and genus level are displayed for each sample at baseline (T1). All sequences were submitted to Qiime for taxonomic identification. Top phyla and genera are displayed. Figures were created in Excel2010.
(TIF)

S1 Table Read loss in classification: 2-day study. (DOCX)

S2 Table Read loss in classification: 9-month study. (DOCX)

S3 Table OTUs_Changed_2Fold. (XLSX)
