# Supplementary figures and images for: Short term dynamics of the sputum microbiome among COPD patients
Source: PLoS One. 2018 Mar 8;13(3):e0191499. doi: 10.1371/journal.pone.0191499 (PMC5843169; doi:10.1371/journal.pone.0191499)

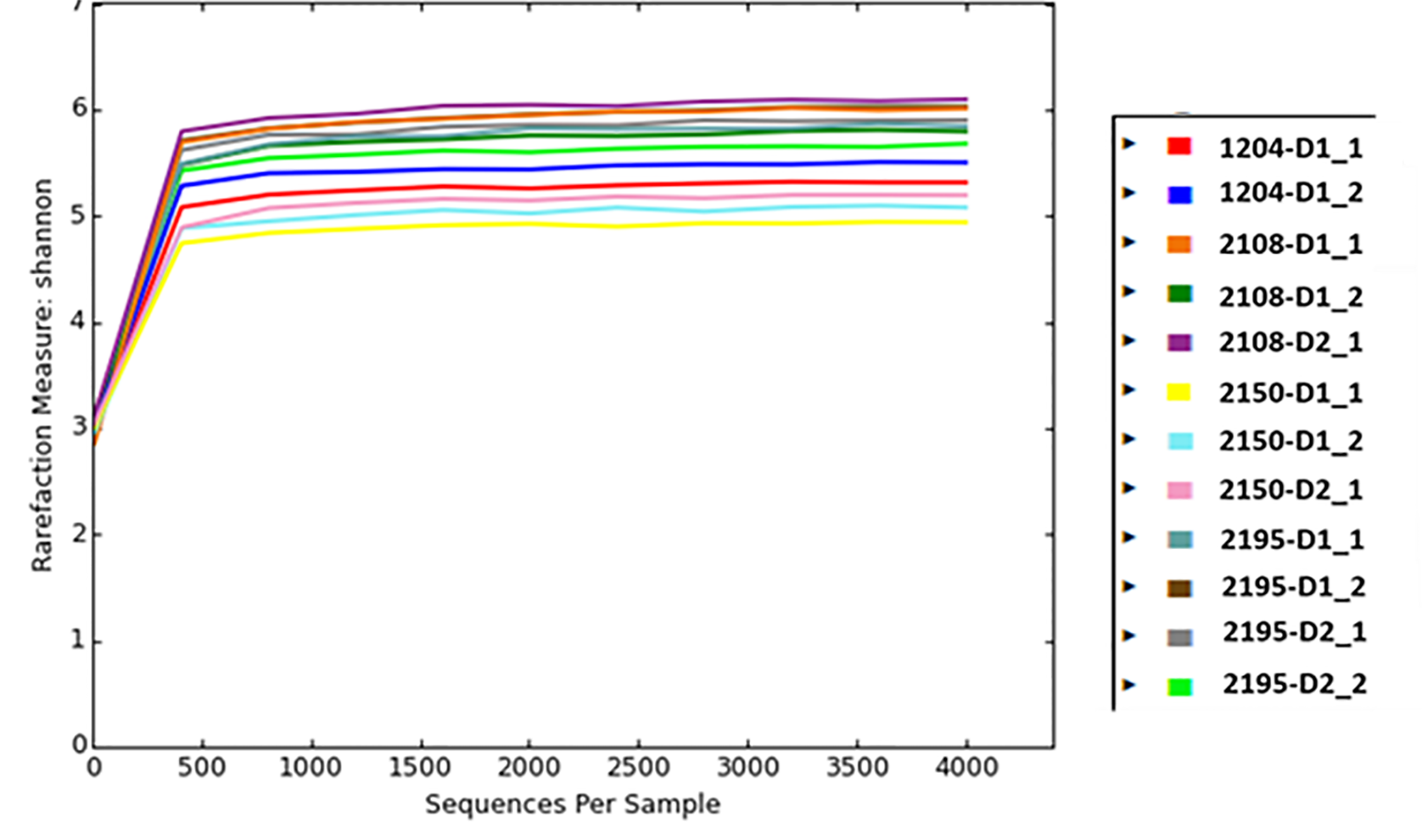

Supplement: S1 Fig — X-axis represents sequences per sample. Y-axis is Shannon rarefraction measure. Legend shows the color representing each sample. Figure was created in Qiime version 1.9.1-dev. (TIF) [file pone.0191499.s001.tif]

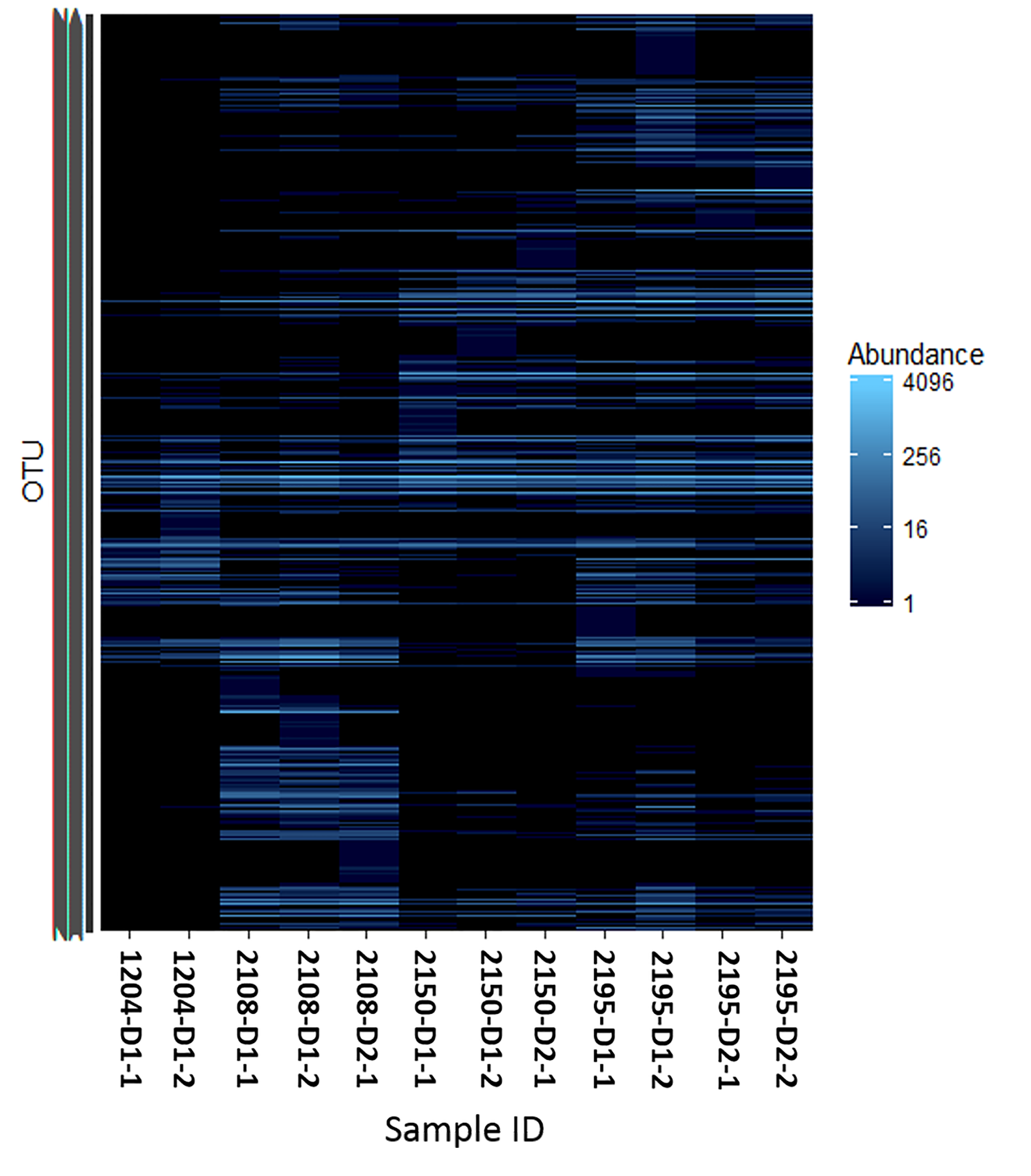

Supplement: S2 Fig — Lighter blue shows greater abundance compared to dark blue/black. Figure was created in R (phyloseq version 1.7.12) using all aligned OTUs. (TIF) [file pone.0191499.s002.tif]

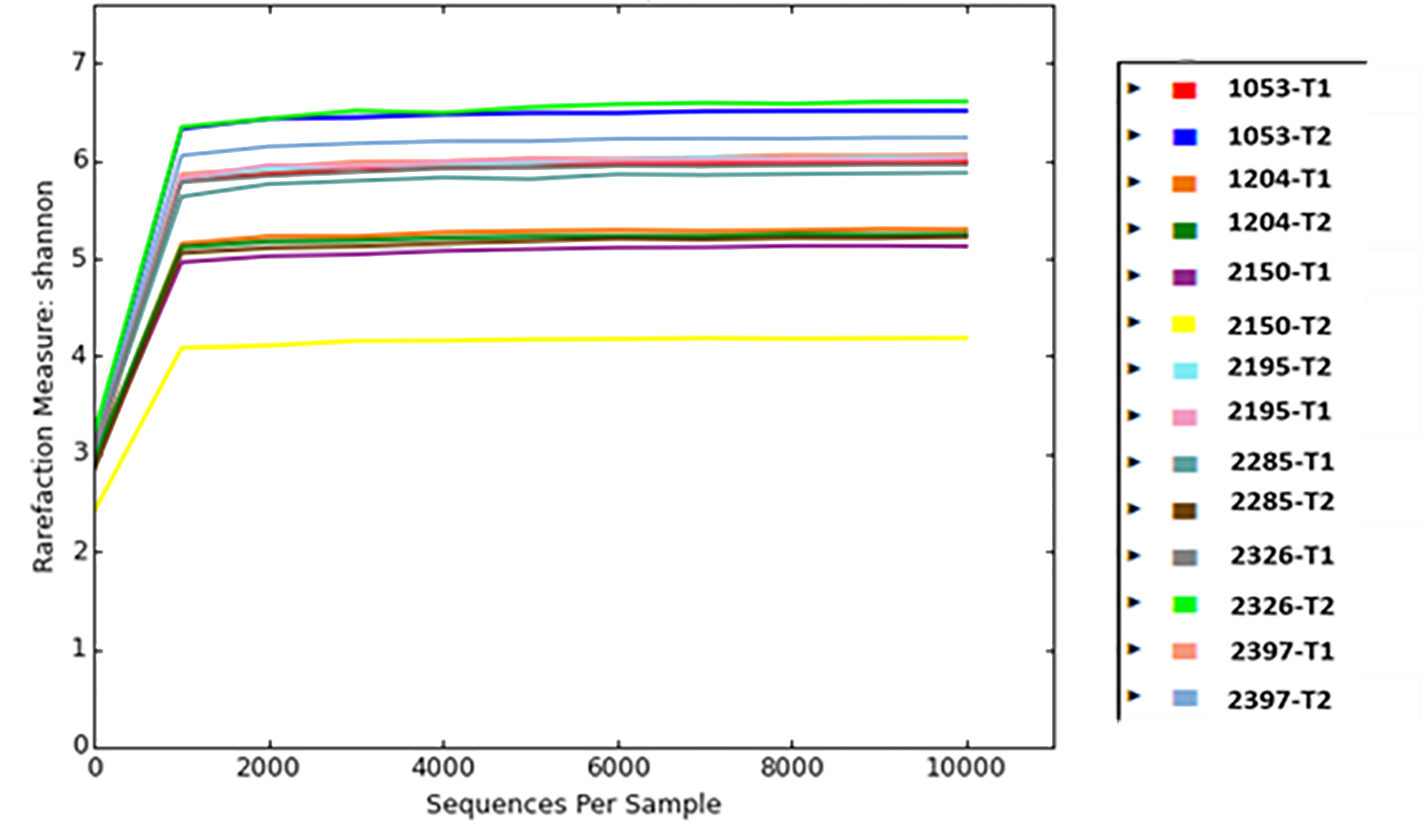

Supplement: S3 Fig — X-axis is sequences per sample. Y-axis is Shannon Rarefraction Measures. Legend shows the color representing each sample. Figure was created in Qiime version 1.9.1-dev. (TIF) [file pone.0191499.s003.tif]

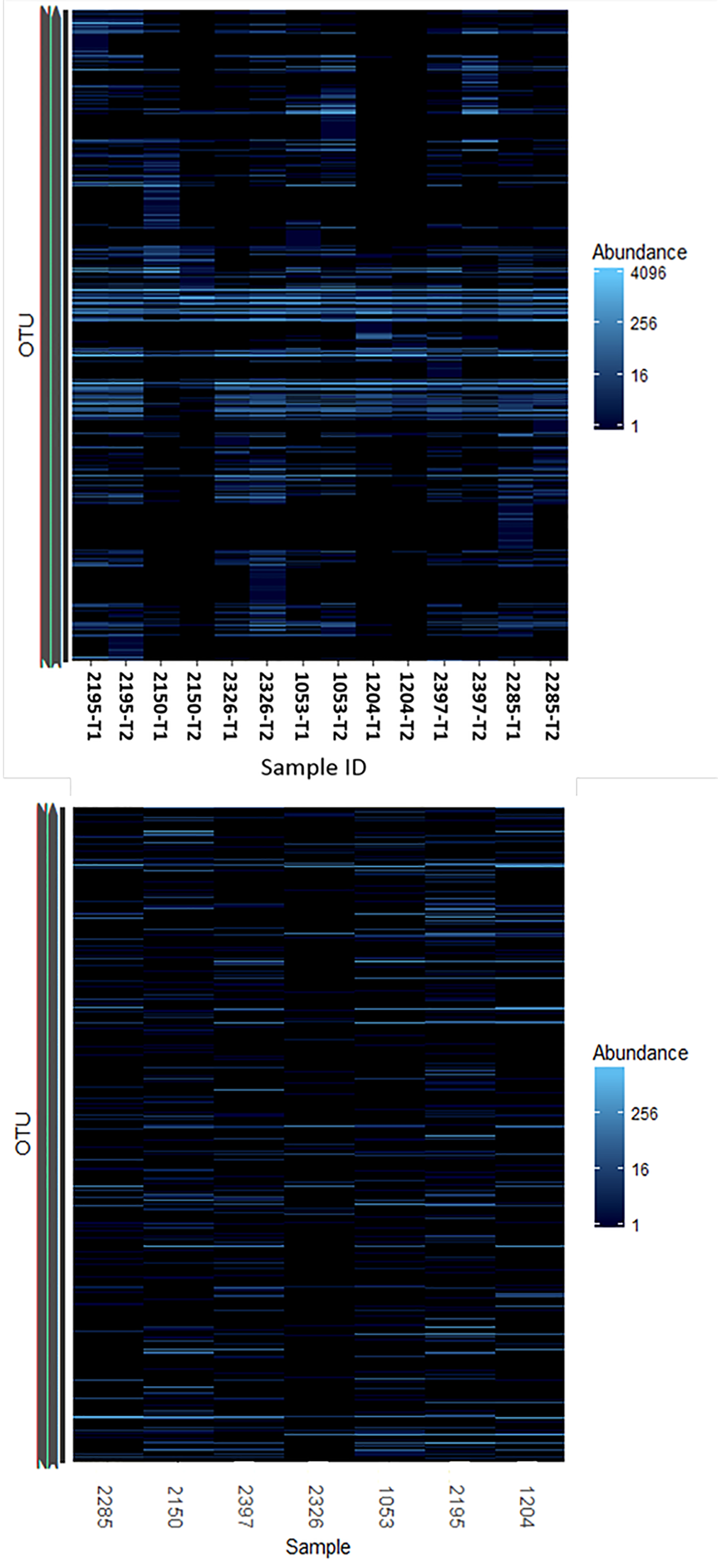

Supplement: S4 Fig — Top. Heat map of OTU abundances at baseline (T1) and at the later time point (T2) in the 9-month Study. Bottom. Heat map of differences of OTU abundances between T1 and T2 (T2 minus T1). Lighter blue shows greater abundance difference compared to dark blue/black. Figures were created in R (phyloseq version 1.7.12) using all aligned OTUs. (TIF) [file pone.0191499.s004.tif]

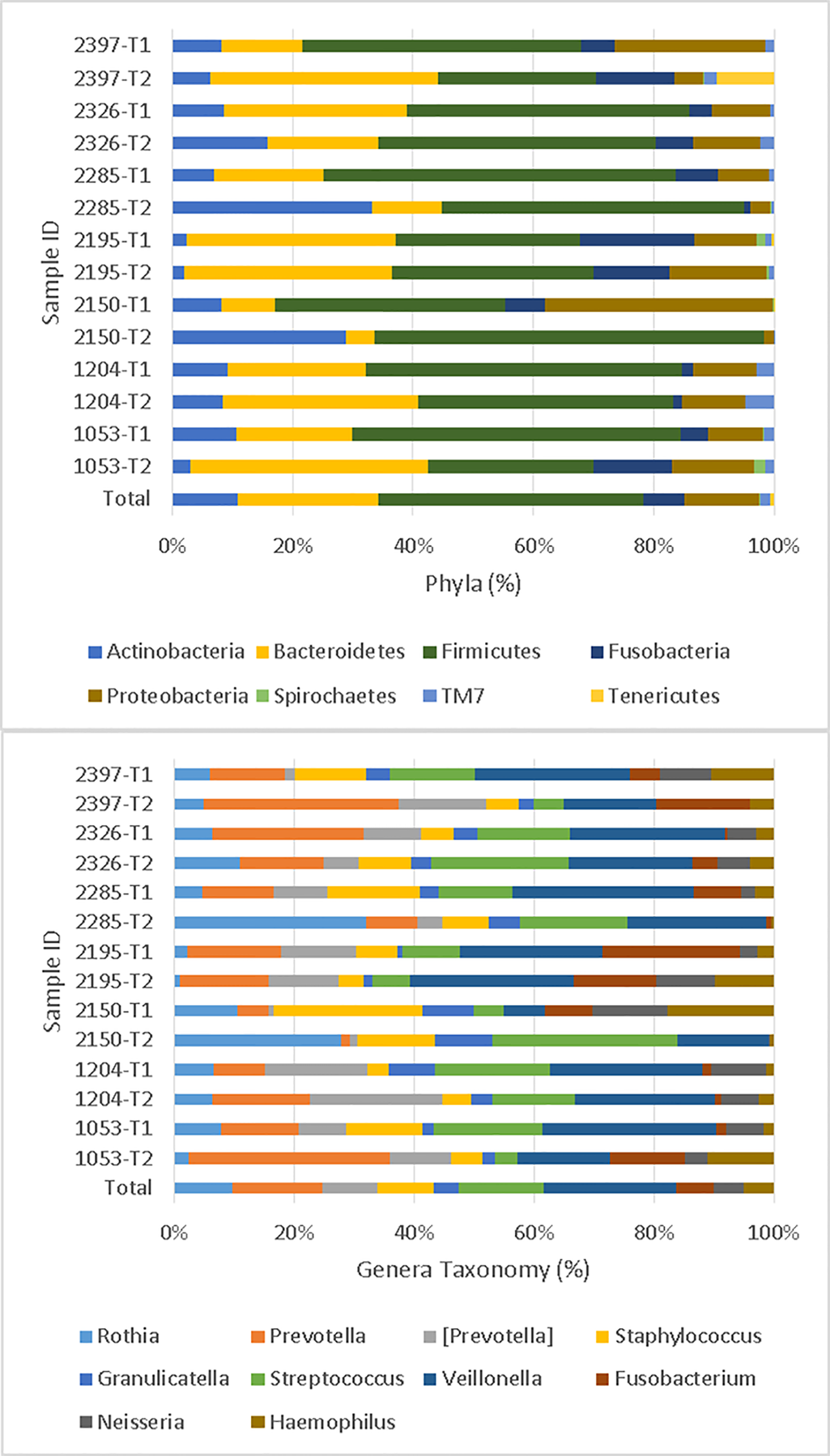

Supplement: S5 Fig — Top. Taxonomic identification at the phylum level in the 9-month study. Bottom. Taxonomic identification at the Genus level. Taxonomic results at the phylum and genus level are displayed for each sample at baseline (T1). All sequences were submitted to Qiime for taxonomic identification. Top phyla and genera are displayed. Figures were created in Excel2010. (TIF) [file pone.0191499.s005.tif]
