# Supplementary material for: Short term dynamics of the sputum microbiome among COPD patients
Source: PLoS One. 2018 Mar 8;13(3):e0191499. doi: 10.1371/journal.pone.0191499 (PMC5843169; doi:10.1371/journal.pone.0191499)
Supplement: S1 Table — (DOCX) [file pone.0191499.s006.docx]

| S1 Table. Read Loss in Classification: 2-Day Study | | |
| --- | --- | --- |
| Sample ID | Initial Reads | Clustered as OTUs |
| 1204-D1_1 | 4213 | 4078 |
| 1204-D1_2 | 13201 | 11289 |
| 2108-D1_1 | 17782 | 17268 |
| 2108-D1_2 | 26469 | 25587 |
| 2108-D2_1 | 17692 | 17153 |
| 2150-D1_1 | 30231 | 16472 |
| 2150-D1_2 | 24614 | 22543 |
| 2150-D2_1 | 26661 | 23225 |
| 2195-D1_1 | 21008 | 18999 |
| 2195-D1_2 | 39694 | 36329 |
| 2195-D2_1 | 15867 | 25397 |
| 2195-D2_2 | 28612 | 14096 |
