# Supplementary material for: Short term dynamics of the sputum microbiome among COPD patients
Source: PLoS One. 2018 Mar 8;13(3):e0191499. doi: 10.1371/journal.pone.0191499 (PMC5843169; doi:10.1371/journal.pone.0191499)
Supplement: S2 Table — (DOCX) [file pone.0191499.s007.docx]

| S2 Table. Read Loss in Classification: 9-Month Study | | |
| --- | --- | --- |
| Sample ID | Initial Reads | Clustered as OTUs |
| 2195-T1 | 41799 | 40133 |
| 2195-T2 | 35728 | 32448 |
| 2150-T1 | 27467 | 25185 |
| 2150-T2 | 10529 | 10056 |
| 2326-T1 | 16832 | 16114 |
| 2326-T2 | 27873 | 26533 |
| 1053-T1 | 24597 | 22705 |
| 1053-T2 | 38684 | 35736 |
| 1204-T1 | 25146 | 24421 |
| 1204-T2 | 11581 | 11117 |
| 2397-T1 | 16841 | 15470 |
| 2397-T2 | 16558 | 15674 |
| 2285-T1 | 19536 | 18763 |
| 2285-T2 | 24956 | 21459 |
